# High-Yield and High-Titer Production of Levulinic Acid from Red Macroalgae via Sulfuric Acid-Catalyzed Hydrolysis: Optimization by Response Surface Methodology and Application to Actual Biomass

**DOI:** 10.4014/jmb.2606.06033

**Published:** 2026-09-18

**Authors:** Im Su Kang, Seon Hui Kim, Jun Hee Jang, Kyoung Heon Kim, Dong Hyun Kim

**Affiliations:** 1 Department of Integrative Biology, Kyungpook National University, Daegu 41556, Republic of Korea; 2 Department of Chemical Engineering, Rowan University, Glassboro, NJ 08028, USA; 3 Department of Biotechnology, Graduate School, Korea University, Seoul 02841, Republic of Korea; 4 Department of Advanced Bioconvergence, Kyungpook National University, Daegu 41566, Republic of Korea; 5 Institute for Basic Science Convergence, Kyungpook National University, Daegu 41566, Republic of Korea

**Keywords:** Levulinic acid, Red macroalgae, Biorefinery, Platform chemical, Response surface methodology, High-solids loading

## Abstract

Levulinic acid (LA) is a versatile bio-based platform chemical with wide-ranging applications in biofuels, solvents, and biodegradable materials. To establish an efficient LA production strategy, red macroalgae were selected as the biomass feedstock due to their high carbohydrate content—particularly galactan, which is rich in 3,6-anhydro-L-galactose (AHG), a sugar that readily converts to LA under acidic conditions. In this study, a response surface methodology (RSM) based on a Box–Behnken design was used to optimize LA production from agar, the primary polysaccharide in red algae. The RSM model predicted an optimum of 172.5°C, 67.4 min, 8% H_2_SO_4_, with a predicted LA yield of 32.45%, and independent experimental validation under these conditions resulted in an LA yield of 33.23%. The validated reaction conditions were subsequently applied to two species of red macroalgae, *Gracilaria verrucosa* and *Gelidium amansii*. At 1% (w/v) biomass loading, carbohydrate-based LA yields of 52.9% and 48.4% were obtained, with corresponding titers of 3.1 and 3.0 g/L, respectively. Increasing the biomass loading to 25% (w/v) increased the LA titers to 49.8 and 48.6 g/L, while the corresponding carbohydrate-based yields decreased to 34.6% and 31.3%, respectively. These findings highlight the potential of high-solids processing as a practical approach for LA production from marine biomass and provide a basis for further process development toward sustainable biorefinery applications.

## Introduction

Global concerns over climate change and the depletion of fossil fuels have intensified research into sustainable alternatives to petroleum-derived feedstocks [1]. Biorefineries, traditionally reliant on fermentable sugars from lignocellulosic biomass, have emerged as promising substitutes for fossil-based refineries. However, sugar-based biorefineries face significant challenges due to the inherent recalcitrance of lignocellulosic biomass, which contains high levels of lignin and crystalline cellulose [2]. These structural complexities necessitate multi-step processing—including chemical pretreatment, enzymatic hydrolysis, and microbial fermentation—resulting in elevated costs and limited commercial viability.

Levulinic acid (LA), identified by the U.S. Department of Energy as a key biomass-derived platform chemical, represents a viable alternative to conventional sugar-based biorefinery processes [3]. LA can be directly synthesized via acid-catalyzed hydrolysis of biomass, bypassing enzymatic saccharification and microbial fermentation. This streamlined approach significantly reduces process complexity and enhances economic feasibility [1]. Structurally, LA is a five-carbon carboxylic acid bearing both ketone and carboxylic acid functional groups, enabling its conversion into a wide array of high-value products, including pharmaceuticals, agrochemicals, biodegradable polymers, solvents, and biofuel [1, 3, 4].

Marine macroalgae have recently gained attention as promising biomass feedstocks due to their rapid growth, high carbon fixation capacity, absence of lignin, and exceptionally high carbohydrate content [5]. Red algae species such as *Gracilaria* and *Gelidium* are particularly attractive, with carbohydrate contents reaching up to 80% of their dry weight [6, 7]. The primary carbohydrate in red algae, agar, is a heterogeneous galactan composed of agarose (70–90%) and agaropectin (10–30%). Agarose is a linear polysaccharide consisting of alternating units of D-galactose and 3,6-anhydro-L-galactose (AHG). Notably, AHG contains an anhydro ring structure that is highly susceptible to acid hydrolysis under elevated temperatures, readily converting into 5-hydroxymethylfurfural (5-HMF)—a key intermediate in LA production [8, 9]. This unique carbohydrate profile enables red algae to serve as an efficient and low-recalcitrance feedstock for LA synthesis.

LA production typically involves acid-catalyzed hydrolysis, proceeding through hexose dehydration to 5-HMF followed by rehydration to LA [4]. In this study, sulfuric acid was selected as the catalyst due to its proven efficacy, cost-effectiveness, and commercial availability. While sulfuric acid is a well-established catalyst, its application in this study led to notable improvements in LA yield and titer. Response surface methodology (RSM) was employed to systematically optimize reaction conditions using agar as a model substrate. The predicted optimum was independently validated using agar and subsequently applied to actual biomass from the red macroalgae *Gracilaria verrucosa* and *Gelidium amansii* to evaluate the transferability of the optimized conditions. To our knowledge, this study reports the high LA yields from red macroalgae under homogeneous acid-catalyzed hydrolysis. Furthermore, high-solids loading of up to 25% (w/v) was successfully implemented, significantly enhancing LA titers and demonstrating the potential of high-solids processing for further process development. Overall, this work provides a basis for the further development of sulfuric acid-catalyzed LA production from marine biomass.

## Materials and Methods

### Materials

D-Galactose, D-glucose (≥ 98%), and sulfuric acid (H_2_SO_4_, 98%) were purchased from Daejung Chemicals (Republic of Korea). Agar was obtained from BD Difco (USA) and Bioneer (Republic of Korea). Red macroalgae species *G. verrucosa* and *G. amansii* were collected from Wando (Republic of Korea) and Jeju Island (Republic of Korea), respectively. The harvested samples were thoroughly washed with tap water to remove surface salts and impurities, then dried at 45°C for 5 d. The dried biomass was ground and sieved to a particle size of less than 1 mm, and stored in sealed plastic bags at -20°C until further use.

### Composition Analysis of Red Macroalgal Biomass

The chemical composition of *G. verrucosa* and *G. amansii* was analyzed to determine their carbohydrate, moisture, ash, and crude protein contents.

Carbohydrate content – specifically glucan and galactan, excluding AHG - was determined using the Laboratory Analytical Procedure (LAP) for structural carbohydrates from NREL [10]. Briefly, 300 mg of dried sample was hydrolyzed with 72% (w/w) sulfuric acid at 30°C for 1 h in a water bath. The mixture was then diluted to 4% acid and subjected to secondary hydrolysis in an autoclave at 121°C for 1 h. After cooling for 30 min, the hydrolysate was neutralized to pH 6 using calcium carbonate. Sugar composition was analyzed using high-performance liquid chromatography (HPLC), and the total carbohydrate content was calculated according to LAP. Since AHG cannot be accurately quantified using this method, its content was measured separately using the resorcinol–HCl colorimetric assay. For this, samples were hydrolyzed in 0.5 M HCl for 180 min and diluted to approximately 4 mg/mL to fall within the AHG calibration range. The resorcinol reagent was freshly prepared by mixing concentrated HCl (12 M, 100 mL) with a resorcinol solution (150 mg in 100 mL; 9 mL) and an acetaldehyde solution (0.04% v/v; 1 mL), and used within 3 h. For the assay, 30 μL of sample (1 mg/mL) was mixed with 200 μL of water and 1.0 mL of resorcinol reagent, chilled on ice for 5 min, incubated at room temperature for 2 min, heated at 80°C for 10 min, and then cooled immediately on ice for another 5 min. Absorbance was measured at 555 nm within 15 min of reaction completion.

Moisture content was measured by oven-drying the sample at 105°C until a constant weight was achieved [11]. The difference in weight before and after drying was used to calculate water content.

Ash content was determined by incinerating the dried sample in a muffle furnace (LEF-130S-2, LabTech, Republic of Korea) at 575°C overnight until a constant weight was achieved [11].

Crude protein content was assessed using the Kjeldahl method. Samples were digested at 420°C using a heating digester (DK20, Velp Scientifica, Italy), neutralized with a KS 1000 Scrubber (Velp Scientifica), and distilled using a semi-automatic distillation unit (UDK 139, Velp Scientifica). The released ammonia was quantified by titration with 0.1 N sulfuric acid.

### Optimization of LA Production from Agar Using RSM

LA production from agar was catalyzed by sulfuric acid in a microwave-assisted digestion system (ETHOS 1, Milestone, Italy) using a 100 mL Teflon vessel with a working volume of 30 mL. The reaction was conducted without mechanical stirring. The agar concentration was fixed at 10 g/L (1%, w/v) for all RSM experiments. Sulfuric acid concentration is expressed as wt% relative to the total reaction mixture. Process optimization was performed using RSM based on a Box–Behnken design to investigate the interactive effects of three key variables: reaction temperature, reaction time, and sulfuric acid concentration. These variables were designated as X_1_, X_2_, and X_3_, respectively, and were investigated over the ranges of 170–190°C, 10–108 min, and 1–8 wt%, respectively. The corresponding coded and actual levels are presented in Table 1, and the experimental design matrix is presented in Table 2.

Experimental design and statistical analyses were conducted using Minitab software (version 21.3, Minitab LLC, USA). The data were fitted to a second-order polynomial model via multiple regression analysis. Model significance and the influence of individual variables were assessed using analysis of variance (ANOVA). Model adequacy was evaluated based on the coefficient of determination (*R*^2^), adjusted *R*^2^, predicted *R*^2^, and lack-of-fit tests.

A total of 15 experimental runs were designed, with each run performed in triplicate, resulting in 45 total observations. The experimental runs are summarized as mean ± standard deviation in Table 2, and the corresponding RSM analysis is presented in Table 3. Post-reaction, samples were cooled, centrifuged at 12,000 rpm for 15 min, and filtered through a 0.2 μm syringe filter. The supernatant was stored at 4°C for further analysis.

### Experimental Validation of the RSM-Predicted Optimum

An independent validation experiment was conducted using agar under the optimal conditions predicted by the RSM model. The predicted optimum of 172.5°C, 67.4 min, and 8 wt% H_2_SO_4_ was experimentally tested using the same microwave-assisted digestion system and analytical procedures described above. LA titer and yield were determined from the experimental product and compared with the corresponding model-predicted values. The validation experiment was performed in triplicate, and the results are reported as mean ± standard deviation.

### Application to Actual Biomass and High-Solids Loading

Following experimental validation of the RSM-predicted optimum using agar, the validated reaction conditions were applied to the red macroalgae *G. verrucosa* and *G. amansii*. Hydrothermal conversion was carried out in the same microwave-assisted digestion system (ETHOS 1, Milestone, Italy) using sulfuric acid as a catalyst. The validated reaction conditions were 172.5°C, 67.4 min, and 8 wt% H_2_SO_4_.

To evaluate the effect of increasing substrate concentration on LA production, high-solids loading experiments were conducted. Two variables were examined: ramping time (time required to reach the target temperature) and substrate concentration. Ramping times of 15, 30, 45, and 60 min were tested, while substrate concentrations were tested at 1%, 5%, 10%, 15%, 20%, and 25% (w/v). Post-reaction, solid residues were removed by centrifugation (12,000 rpm, 15 min), followed by filtration through a 0.2 μm membrane. The resulting supernatant was used for the quantification of LA and other by-products. All high-solids loading experiments were performed in triplicate, and the data are presented as the mean ± standard deviation (SD).

### Product Analysis

Quantification of sugars (galactose and glucose), LA, and byproducts (5-HMF and formic acid (FA)) was performed using HPLC (Agilent 1260 Infinity II, USA) equipped with a refractive index detector (RID; Agilent 1260 Infinity II) and a Hi-Plex H column (300 × 7.7 mm; Agilent, USA). The column was maintained at 65°C, with 0.0085 M sulfuric acid (H_2_SO_4_) as the mobile phase at a flow rate of 0.6 mL/min. LA yield was calculated using two approaches to evaluate process efficiency. The first method expresses yield based on the total substrate weight (Y_s_), while the second method calculates yield relative to the carbohydrate content of the red macroalgal biomass (Y_c_). These calculations are shown in Eqs. (1) and (2):

$${\text{Y}}_{\text{s}}\left(\text{\%}\right)=\frac{\text{Final LA concentration (g/L) in the hydrolysate}}{\text{Input substrate concentration (g/L)}}\times 100$$ (1)

$${\text{Y}}_{\text{c}}\left(\text{\%}\right)=\frac{\text{Final LA concentration (g/L) in the hydrolysate}}{\text{Input carbohydrate concentration (g/L) in red algae}}\times 100$$ (2)

## Results and Discussion

### Optimization of LA Production from Agar Using RSM

Red macroalgae, such as *G. verrucosa* and *G. amansii*, are rich in carbohydrates, primarily in the form of agar, which consists of agarose and agaropectin [6, 7]. The dominant sugar units in these galactan polymers are D-galactose and AHG. Unlike terrestrial biomass, which contains recalcitrant lignin and crystalline cellulose, red macroalgae possess relatively simple carbohydrate structures and are devoid of lignin [7]. This structural simplicity allows for more efficient hydrolysis under milder conditions, making red macroalgal carbohydrates more favorable for conversion into fermentable sugars.

However, the transformation of red macroalgal carbohydrates into LA still involves a multi-step chemical pathway: hydrolysis of polysaccharides into monomeric sugars, dehydration into 5-HMF, and subsequent rehydration to produce LA [1, 12]. Sulfuric acid, a cost-effective and industrially established Brønsted acid, is widely used to catalyze these reactions in homogeneous systems [13, 14]. In particular, sulfuric acid effectively promotes the conversion of AHG into 5-HMF due to the inherent instability of its anhydro ring under acidic and high-temperature conditions [8].

In this study, RSM was employed to optimize LA production from agar, the predominant polysaccharide in red macroalgae, using sulfuric acid as a homogeneous acid catalyst. The goal was to identify key operational parameters and their interactions to establish an efficient conversion strategy applicable to whole macroalgal biomass. A Box–Behnken design was constructed using three independent variables: reaction temperature (170–190°C), reaction time (10–108 min), and sulfuric acid concentration (1–8%, w/w). The coded and actual levels of these variables are listed in Table 1. A total of 15 experimental runs were designed, with each run performed in triplicate (45 total observations), and the corresponding mean LA titer and yield are presented in Table 2.

LA yield varied considerably depending on reaction conditions, ranging from 12.5% to 34.8%. The highest LA yield (34.8%) was achieved under 180°C, 59 min, and 4.5% H_2_SO_4_, which also produced the highest LA titer of 3.5 g/L. In contrast, the lowest yield (12.5%) was obtained at 180°C, 10 min, and 1% acid. Catalyst concentration was identified as the most influential factor, followed by reaction time, while temperature had a comparatively lesser effect (Fig. 1). LA yields below 20% were generally associated with low acid concentrations (1%) or short reaction times (10 min), emphasizing the importance of sufficient catalyst loading and residence time for effective conversion.

Based on the fitted regression model, the predicted optimal conditions for LA production were 67.4 min reaction time, 172.5°C temperature, and 8.0% sulfuric acid (Table S1). The second-order polynomial model for LA yield (*Y*_LA_) is given by Eq. (3):

$$Y_{\text{LA}}=30.233+1.208X_{1}+2.980X_{2}+4.620X_{3}-1.912X_{1}X_{2}-1.509X_{1}X_{3}-3.283X_{2}X_{3}-0.417X_{1}^{2}-3.378X_{2}^{2}-2.492X_{3}^{2}$$ (3)

where X_1_, X_2_, and X_3_ represent the coded values for reaction temperature, reaction time, and acid concentration, respectively.

The statistical significance and model fit are summarized in Table 3. The model yielded an *F*-value of 14.46 and a *p*-value < 0.001, indicating strong statistical significance. The model showed a good overall fit, with an *R*^2^ value of 0.788 and a non-significant lack-of-fit (p = 0.198), supporting the adequacy of the fitted model. As shown in the standardized Pareto chart (Fig. 1), sulfuric acid concentration (X_3_) had the most significant positive effect on LA yield, followed by reaction time (X_2_), while reaction temperature (X_1_) had the least impact. Among the quadratic terms, the coefficients of X_1_^2^, X_2_^2^, and X_3_^2^ were negative, indicating downward curvature in the response surface. The quadratic effects of reaction time (X_2_^2^, p = 0.001) and H_2_SO_4_ concentration (X_3_^2^, p = 0.008) were statistically significant, whereas the quadratic effect of temperature (X_1_^2^, p = 0.642) was not. The significant negative quadratic effect of H_2_SO_4_ concentration suggests that excessive acid loading may decrease LA yield, potentially due to secondary reactions such as humin formation [15, 16].

A previous study reported an LA yield of up to 45.8% based on input agarose under identical reaction conditions (180°C, 10 min, 1% H_2_SO_4_) using a microwave-assisted system [17]. This discrepancy may be attributed to the use of agarose—a more refined polysaccharide with a higher AHG content that readily undergoes acid-catalyzed dehydration to 5-HMF and subsequent rehydration to LA. In contrast, the agar used in this study contains both agarose and agaropectin, the latter of which includes various sulfated functional groups that may hinder efficient conversion. While feedstock purity influences conversion efficiency, using less-refined polysaccharides offers economic benefits. Notably, the present study achieved a substantial LA yield (34.8%) from agar by systematically optimizing key reaction parameters, demonstrating the feasibility of using lower-purity feedstocks for further development of red macroalgae-based biorefinery processes.

### Experimental Validation of the RSM-Predicted Optimum

To assess the predictive reliability of the RSM model, an independent validation experiment was performed using agar under the predicted optimal conditions of 172.5°C, 67.4 min, and 8.0 wt% H_2_SO_4_. The model predicted an LA yield of 32.45% and a corresponding LA titer of 3.24 g/L, whereas the experimentally observed LA yield and LA titer were 33.23 ± 0.80% and 3.32 ± 0.08 g/L (n = 3), respectively. The difference between the predicted and experimental LA yields was 0.78 percentage points, corresponding to a relative deviation of approximately 2.4%. The close agreement between the predicted and experimentally observed LA yields demonstrates the predictive reliability of the RSM model. Therefore, the validated optimal conditions were subsequently applied to actual red macroalgal biomass.

### Reciprocal Effects between Reaction Factors on LA Production

To elucidate the interactive effects among temperature, catalyst concentration, and reaction time on LA production from agar, three-dimensional response surface plots and corresponding contour plots were analyzed. Fig. 2(A–I) illustrate the impact of varying two parameters while keeping the third constant, enabling visual assessment of reciprocal relationships among the three factors . Overall, LA yield increased with longer reaction times and higher temperatures or catalyst concentrations; however, excessive levels of any factor occasionally reduced yield, likely due to thermal degradation or by-product formation.

Fig. 2(A–C) illustrate the effects of reaction time (10–108 min) and temperature (170–190°C) at fixed catalyst concentrations of 1%, 4.5%, and 8%. At 1% catalyst loading (Fig. 2A), LA yield gradually increased with rising reaction temperature and time. With 4.5% catalyst (Fig. 2B), yields exceeded 30% when the reaction time surpassed 75 min and the temperature exceeded 180°C. At 8% catalyst loading (Fig. 2C), even moderate conditions (60–80 min, <180°C) resulted in yields above 30%, highlighting the strong catalytic effect of higher acid concentrations.

Fig. 2D–2F illustrate the interaction between temperature (170–190°C) and catalyst concentration (1–8%) at fixed reaction times of 10, 59, and 108 min. At 10 min (Fig. 2D), LA yield remained below 30% across most conditions, indicating insufficient residence time. At 59 min (Fig. 2E), LA yield improved significantly with increasing temperature and acid concentration, surpassing 30% when both parameters exceeded 180°C and 6%, respectively. In contrast, Fig. 2F shows that while moderate acid concentrations (4–6%) at ~180°C supported high LA yields of approximately 35%, further increases in acid loading reduced the yield—especially at elevated temperatures—likely due to side reactions such as humin formation [15].

Fig. 2(G–I) depict the effects of reaction time (10–108 min) and catalyst concentration (1–8%) at fixed temperatures of 170, 180, and 190°C. At 170°C (Fig. 2G), yields above 30% were achieved when reaction time exceeded 90 min and acid concentration was above 6%, suggesting that sufficient residence time and acid loading can compensate for lower thermal input. At 180°C (Fig. 2H), both longer reaction times and higher acid concentrations positively influenced LA yield, with values exceeding 30% when reaction time surpassed 75 min and acid concentration exceeded 5%. At 190°C (Fig. 2I), although high LA yields (>30%) were maintained under prolonged reaction and elevated acid concentrations, the overall response surface plateaued, indicating diminishing returns and potential degradation of intermediates at excessive thermal exposure.

These findings confirm that, among the test parameters, catalyst concentration and reaction time exert the most pronounced influence on LA yield, consistent with the Pareto chart (Fig. 1) and ANOVA results (Table 3). Among the interaction terms, temperature × time (X_1_X_2_) and time × catalyst (X_2_X_3_) were statistically significant, whereas temperature × catalyst (X_1_X_3_) was not statistically significant. While temperature alone was not the dominant factor, it played a critical role in modulating polysaccharide reactivity when paired with appropriate catalyst loading and residence time.

In summary, the response surface analysis demonstrates that LA yield from agar is strongly influenced by the interaction between catalyst concentration and reaction time. Although temperature was not the most influential factor when considered independently, it significantly enhanced conversion efficiency when combined with optimized catalyst loading and reaction time. These insights establish a robust framework for selecting suitable reaction conditions. The predicted optimum was subsequently subjected to an independent experimental validation using agar, and the validated conditions were then applied to actual red macroalgal biomass.

### Application of the Model to Actual Biomass

Following the experimental validation of the RSM-predicted optimum using agar, two commercially important red macroalgae with high agar content — *G. verrucosa* and *G. amansii* —were selected for their widespread use in agar extraction and their agarose-rich composition, consisting of D-galactose and L-AHG. To confirm their suitability, a compositional analysis was conducted for both species. As shown in Table 4, G. verrucosa contained 38.53% galactan, 9.76% glucan and 9.29% AHG while *G. amansii* contained 27.25% galactan, 19.46% glucan and 15.38% AHG based on dry weight. In addition to galactan and AHG, the significant glucan content in both species enhances their potential for LA production, offering a broader carbohydrate base than agar alone.

The optimized reaction conditions—172.5°C, 67.4 min, 8% H_2_SO_4_—derived from agar-based modeling, were subsequently applied to both macroalgal biomasses. At the 1% (w/v) substrate loading used for direct application of the validated conditions, LA yields reached 52.9% (*Y_c_*) and 30.5% (*Y_s_*) for *G. verrucosa*, and 48.4% (*Y_c_*) and 30.1% (*Y_s_*) for *G. amansii*, representing some of the highest reported yields from red macroalgae using a conventional homogeneous catalyst (Fig. 3 and Table 5).

To contextualize these results, a comparison was made with previously reported LA yields from red macroalgae (Table 5). Previous studies have reported maximum carbohydrate-based LA yields (*Y_c_*) of 36.9% for *G. verrucosa* and 37.6% for *G. amansii* under their respective optimized conditions [18, 19]. A more recent study reported a yield of 30.3% from *G. verrucosa* using 6.25% Purolite CT269DR, a costly commercial ionic resin catalyst [20].

These findings demonstrate that the RSM-derived conditions optimized using agar can be effectively transferred to actual red macroalgal biomass, supporting the practical applicability of the optimized process. However, because the optimization was performed using agar as a model substrate rather than directly on actual biomass, the current results are interpreted as showing that the agar-based optimized conditions can be successfully transferred to actual red macroalgal biomass, rather than demonstrating that the biomass itself has been fully optimized. Because actual macroalgal biomass contains additional components, such as glucan, ash, and proteins, further optimization using actual biomass may improve LA production.

### Effect of High-Solids Loading on LA Production

For future scale-up of LA production, achieving a high product titer is essential to reduce downstream processing costs and improve overall process economics. Increasing substrate concentration is a direct strategy to enhance titer while minimizing energy and water input per unit of product. However, high-solids systems often face challenges such as elevated viscosity, impaired mass and heat transfer, and non-uniform temperature gradients. To address these issues, the feasibility of LA production from *G. verrucosa* and *G. amansii* was evaluated under high-solids loading conditions, with substrate concentrations increased up to 25% (w/v) (Fig. 3). Additionally, the effect of temperature ramping time on LA yield consistency was investigated to mitigate thermal degradation and promote uniform heat distribution during microwave-assisted hydrolysis.

At a fixed biomass concentration of 5% (w/v), ramping times of 15, 30, 45, and 60 min were tested (Fig. S1). Short ramping times resulted in variable LA yields, likely due to uneven thermal distribution. In contrast, a ramping time of 60 min led to stable and reproducible LA yields, indicating that gradual temperature elevation is critical for maintaining efficient hydrolysis and suppressing the formation of undesired by-products under microwave heating.

Subsequently, substrate concentrations ranging from 1% to 25% were tested under optimized reaction conditions and a ramping time of 60 min. As shown in Fig. 3, LA yields remained stable or slightly decreased with increasing solids loading. Notably, even at 20% solids, LA titers reached 42.9 g/L (*Y_c_* = 37.3%) for *G. verrucosa* and 36.3 g/L (*Y_c_* = 29.2%) for *G. amansii*. The highest LA titers for *G. verrucosa* and *G. amansii*—49.8 g/L (*Y_c_* = 34.6%) and 48.6 g/L (*Y_c_* = 31.3%), respectively—were achieved at 25% solids (Fig. 3). To our knowledge, these are among the highest reported LA titers from seaweed-derived biomass using homogeneous acid catalysis (Table 5).

This performance is further supported by literature comparisons (Table 5). Previous studies reported LA titers of 22.0 g/L from *G. verrucosa* using methanesulfonic acid [19], and 22.6 g/L using 6.25% Purolite CT269DR, a commercial ionic resin (Park & Jeong, 2024). In contrast, our study more than doubled these values using only sulfuric acid and red macroalgae as feedstock. Additionally, when *Eucheuma spinosum*, a red macroalga commonly used for iota-carrageenan extraction, was used as the feedstock, only 4.3 g/L of LA was obtained at 5% substrate concentration, corresponding to a low yield of 11.6% [21]. *Eucheuma spinosum* is known to possess a high sulfate content and low proportion of AHG, both of which contribute to a weaker gel structure and reduced reactivity under acid-catalyzed hydrolysis [22]. In contrast, at the same substrate loading of 5% (w/v), our study achieved 11.2 g/L from *G. verrucosa* and 10.8 g/L from *G. amansii*, demonstrating higher LA titers than that reported for *E. spinosum*.

Microalgae have also been explored for LA production. For example, *Scenedesmus obliquus* has been investigated using various acid catalysts, including CH_3_SO_3_H, HCl, and H_2_SO_4_ (Table 5). A titer of 16.8 g/L was reported using methanesulfonic acid (CH_3_SO_3_H) at a biomass loading of 12.5% (w/v) [23], which remains lower than the titers achieved in our study from red macroalgae—20.4 g/L from *G. verrucosa* and 19.5 g/L from *G. amansii* —even at a lower biomass concentration of 10% (w/v).

Furthermore, when compared to lignocellulosic biomass such as corncob, one of the most extensively studied feedstocks for LA production, the contrast is striking. A maximum titer of only 17.2 g/L was achieved under 1 M H_2_SO_4_ at a substrate concentration of 120 g/L (equivalent to 12% w/v)[24]. An LA titer of 18.4 g/L has been reported from poplar wood using 0.95 mol/L *p*-toluenesulfonic acid (*p*-TsOH) at 162°C for 64 min [25]. This value remains lower than the values achieved in our study.

These results highlight the superior reactivity and carbohydrate accessibility of red macroalgal biomass, emphasizing its advantages over lignocellulosic substrates in achieving high LA titers without extensive pretreatment. Our optimized hydrothermal process achieved high LA titers for seaweed-derived feedstocks that are competitive with those reported for terrestrial biomass systems, using a single-step acid-catalyzed reaction. Unlike lignocellulosic biomass, which requires severe pretreatment steps, including lignin removal and enzymatic hydrolysis, red macroalgae lack lignin and are rich in readily hydrolysable galactan and glucan fractions, enabling more efficient and direct LA production even at high biomass concentrations.

Taken together, these findings demonstrate the effectiveness of high-solids loading for achieving high LA titers from red macroalgae. Although a slight decline in yield was observed at the highest substrate concentrations—likely due to increased viscosity and limited mass transfer—the overall conversion remained above 30% in all cases. Importantly, the carbohydrate-rich nature of these red algae, particularly their substantial glucan content (*e.g.*, 9.76% in *G. verrucosa*, 19.46% in *G. amansii*) and AHG content (*e.g.*, 9.29% in *G. verrucosa*, 15.38% in *G. amansii*), supported sustained LA productivity even at elevated substrate concentrations. This underscores the potential of red macroalgal biomass for efficient high-solids chemical conversion and provides a basis for further process development toward industrial application.

### Mass Balance for LA Production from Red Macroalgae

To assess the conversion efficiency of red macroalgal biomass into LA, a mass balance analysis was performed using 100 g of dry biomass from *G. verrucosa* and *G. amansii* under optimized reaction conditions (8% H_2_SO_4_, 172.5°C, 67.4 min; ramping time of 60 min) at a solids loading of 25% (w/v). As illustrated in Fig. 4, both species exhibited similar conversion profiles and comparable performance.

The total carbohydrate content was 57.6 g for *G. verrucosa* and 62.1 g for *G. amansii*, predominantly consisting of galactan (38.5 g and 27.3 g, respectively) and glucan (9.8 g and 19.5 g, respectively), and AHG (9.3 g and 15.4 g, respectively). For theoretical yield calculations, agar was considered as galactan—the representative polysaccharide unit of agarose—consistent with previous studies [26]. Galactan was assumed to hydrolyze into galactose, which then undergoes dehydration and rehydration reactions to form LA and FA. Based on stoichiometric conversion, 1 g of hexose (*e.g.*, galactose or glucose) yields 0.6445 g of LA, derived from the molecular weight ratio of hexose (180.16 g/mol) to LA (116.11 g/mol). In addition, 1 g of AHG yields 0.7161 g of LA, derived from the molecular weight ratio of AHG (162.14 g/mol) to LA (116.11 g/mol). Using this framework, the theoretical LA yields were estimated at 37.9 g for *G. verrucosa* and 41.2 g for *G. amansii*. Under optimized conditions, actual LA production reached 19.9 g and 19.4 g, corresponding to 52.5% and 47.1% of the theoretical maximum, respectively.

FA, which is co-produced during the conversion of 5-HMF to LA in a 1:1 molar ratio, was also quantified. FA yields were 8.4 g for *G. verrucosa* and 7.1 g for *G. amansii*, aligning well with the expected stoichiometry. Residual sugars were detected in minor quantities: galactose (1.0 g and 3.8 g) and glucose (2.1 g and 3.7 g) for *G. verrucosa* and *G. amansii*, respectively. No 5-HMF was detected, and humins were not quantitatively analyzed due to high variability.

Although *G. verrucosa* exhibited slightly higher conversion efficiency—likely attributable to its greater galactan and AHG contents—both species demonstrated robust and efficient carbohydrate-to-LA conversion. These results affirm the versatility and effectiveness of the optimized hydrothermal process across diverse multiple red macroalgal feedstocks with varying carbohydrate compositions.

## Conclusion

This study presents a systematic strategy for LA production from red macroalgal biomass using a sequential optimization and validation approach. Initial process development was carried out using agar—a galactan-rich polysaccharide representative of red algae—through RSM. The predicted optimum of 172.5°C, 67.4 min, and 8% H_2_SO_4_, corresponding to a predicted LA yield of 32.45%, was independently validated using agar, yielding an experimental LA yield of 33.23%. Application of the agar-validated conditions revealed a clear loading-dependent trade-off between LA yield and titer. At 1% (w/v) biomass loading, *G. verrucosa* and *G. amansii* achieved the highest carbohydrate-based LA yields (*Y_c_*) of 52.9% and 48.4%, respectively, with corresponding LA titers of 3.1 and 3.0 g/L. Increasing the biomass loading to 25% (w/v) raised the LA titers to 49.8 and 48.6 g/L, respectively, whereas *Y_c_* decreased to 34.6% and 31.3%. Thus, low substrate loading favored conversion efficiency, whereas high-solids loading increased product concentration while retaining substantial carbohydrate conversion. These titers are competitive with previously reported values for seaweed-derived biomass using a single-step homogeneous acid-catalyzed hydrothermal process (Table 5). The superior reactivity and carbohydrate accessibility of red macroalgae—particularly their galactan and glucan content—enabled efficient conversion without the need for extensive pretreatment steps typically required for terrestrial biomass. Overall, this work demonstrates the potential of red macroalgae as a renewable feedstock for LA production under high-solids loading conditions, providing a basis for the further development of sustainable biorefinery processes. However, this study was conducted using a microwave-assisted batch reactor, and additional investigations are required to assess its industrial-scale feasibility. Future studies should address scale-up beyond microwave-assisted batch operation by evaluating reactor design, heat transfer under high-solids conditions, acid recovery and recycling, corrosion resistance, salt generation during neutralization, downstream LA separation, and solid residue management.

## Supplemental Materials

Supplementary data for this paper are available on-line only at http://jmb.or.kr.



## Figures and Tables

**Fig. 1:**
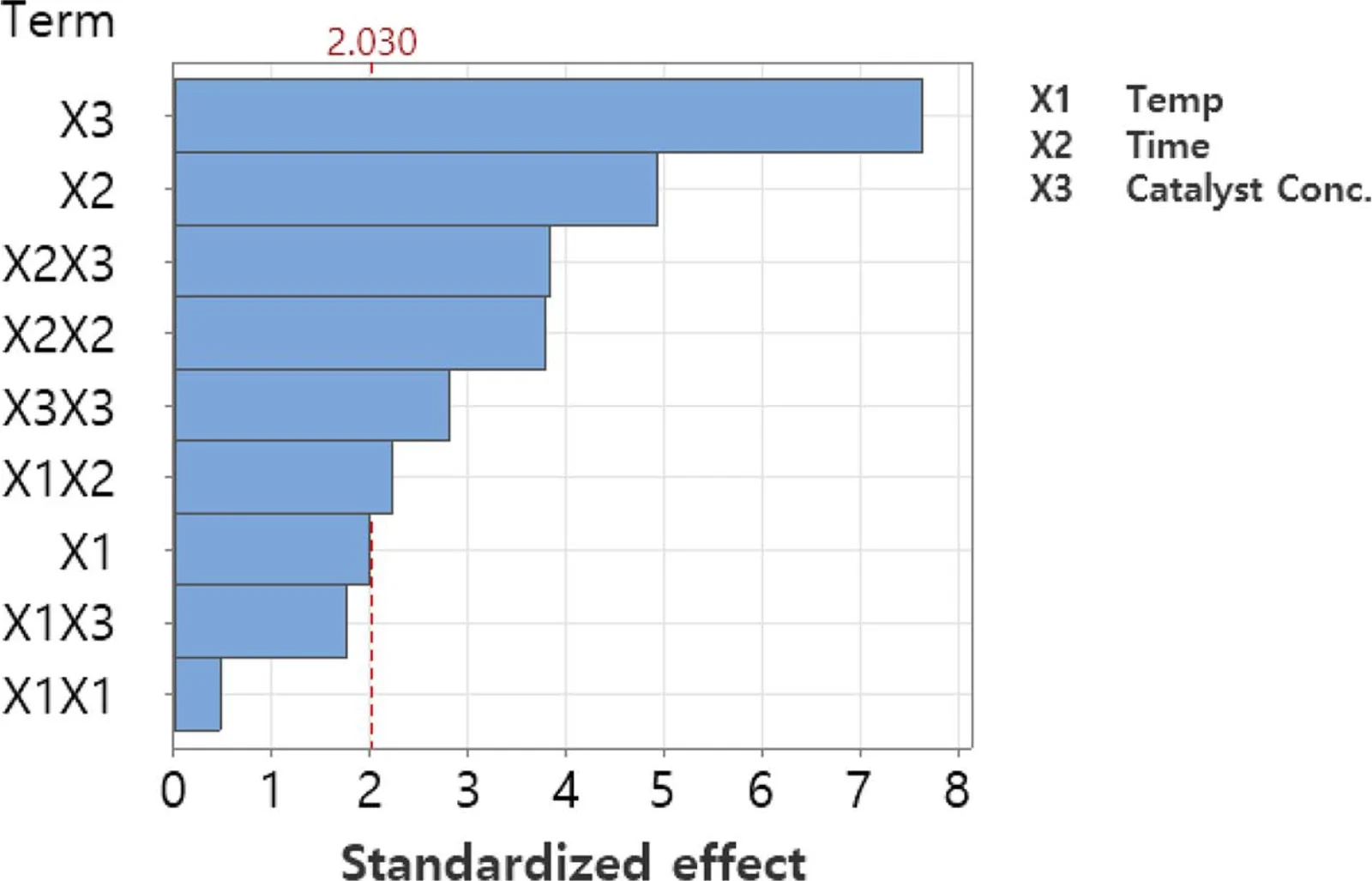
Standardized Pareto chart illustrating the relative influence of main factors, quadratic terms, and interaction effects on LA yield from agar. The vertical dashed line indicates the threshold for statistical significance (α = 0.05).

**Fig. 2:**
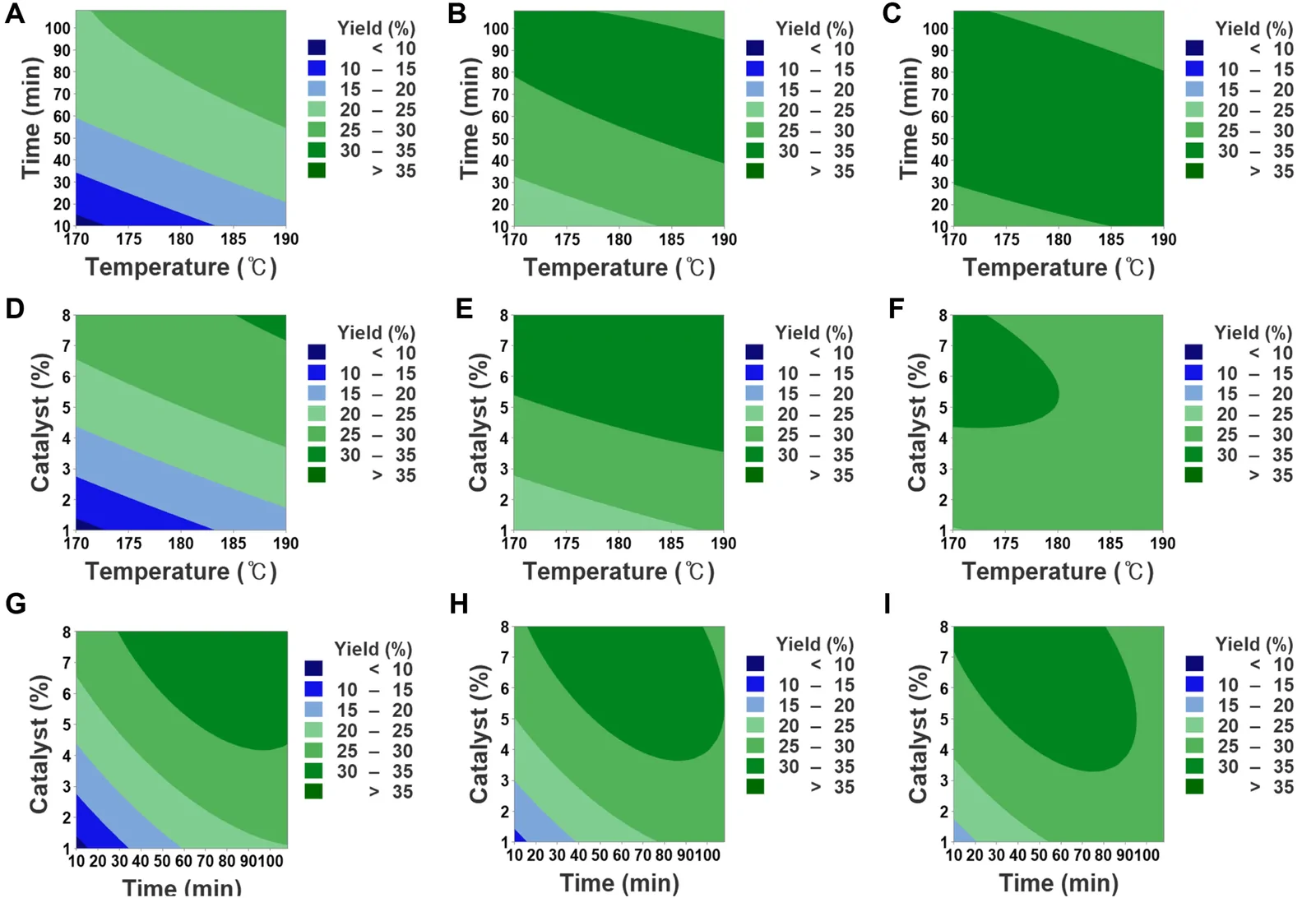
Contour plots showing the interactive effects of reaction parameters on LA yield from agar. (**A–C**) Effect of time and temperature at fixed catalyst concentrations (1%, 4.5%, and 8%); (**D–F**) Effect of temperature and catalyst concentration at fixed reaction times (10, 59, and 108 min); (**G–I**) Effect of time and catalyst concentration at fixed temperatures (170, 180, and 190°C).

**Fig. 3:**
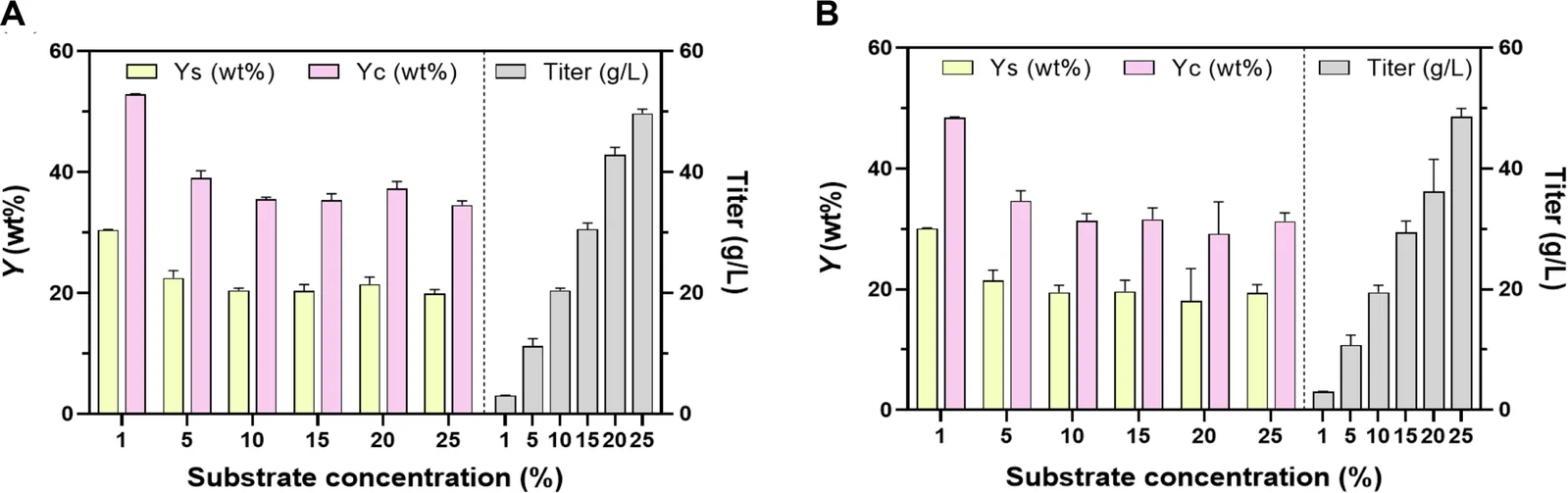
Effect of increasing substrate concentration on LA production from red macroalgae. (**A**) *Gracilaria verrucosa* and (**B**) *Gelidium amansii*. Error bars represent the standard deviation (SD) from three independent experiments. Ys: total-substrate-based yield, Yc: carbohydrate-based yield.

**Fig. 4:**
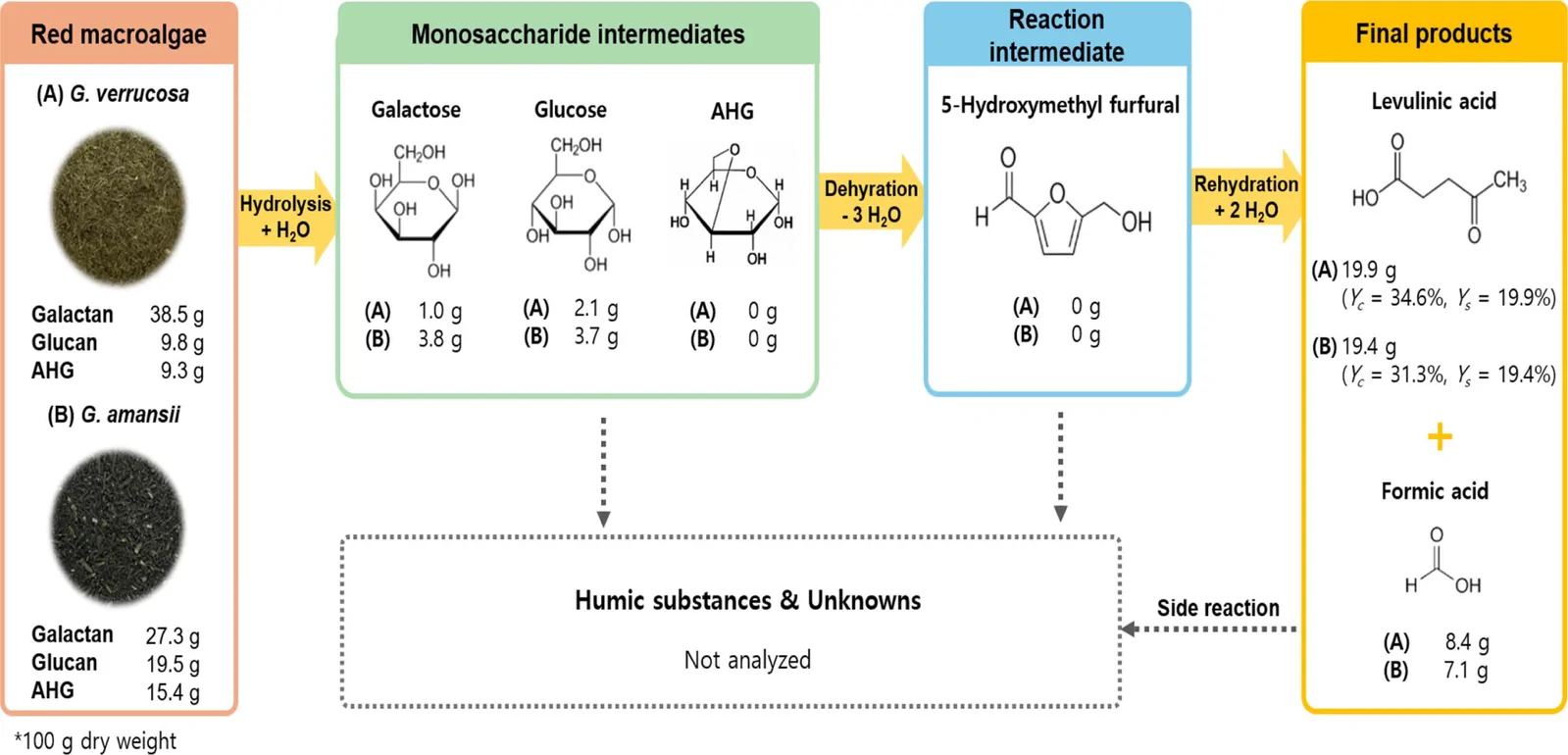
Mass balance analysis of LA production from red macroalgae under optimized conditions (172.5°C, 67.4 min, 8% H_2_SO_4_) at 25% (w/v) substrate concentration. The mass balance is presented on a basis of 100 g dry biomass of (**A**) *Gracilaria verrucosa* and (**B**) *Gelidium amansii*. The conversion pathway includes hydrolysis, dehydration of hexoses to 5-HMF, and subsequent rehydration to LA and FA.

**Table 1:** Coded and actual levels of independent variables in the Box–Behnken design.

Factor	Symbol	Coded level		
		-1	0	+1
Temperature (°C)	X_1_	170	180	190
Time (min)	X_2_	10	59	108
H_2_SO_4_ (wt%)	X_3_	1.0	4.5	8.0

**Table 2:** Experimental design matrix and observed levulinic acid titers and yields from agar using Box–Behnken design.

Temp. (°C)	Time (min)	Catalyst (wt%)	LA titer (g/L)	LA yield (*Y*_*s*_, wt%)
180	10	1	1.3 ± 0.4	12.5
170	59	8	3.1 ± 0.0	30.7
170	108	4.5	3.1 ± 0.2	30.6
180	10	8	3.1 ± 0.3	30.8
170	10	4.5	2.0 ± 0.2	20.4
190	59	8	3.1 ± 0.1	30.7
190	59	1	2.7 ± 0.0	27.0
180	59	4.5	2.8 ± 0.1	27.6
180	59	4.5	3.5 ± 0.2	34.8
180	59	4.5	2.8 ± 0.0	28.3
170	59	1	2.1 ± 0.2	21.0
180	108	1	2.5 ± 0.2	24.5
190	108	4.5	2.9 ± 0.1	28.7
190	10	4.5	2.6 ± 0.4	26.1
180	108	8	3.0 ± 0.0	29.7

Note: values are expressed as mean ± standard deviation of three replicates.

**Table 3:** Analysis of variance (ANOVA) for the fitted response surface model of LA yield from agar.

Source	df	Sum of squares	Mean square	*F*-value	*p*-value
Model	9	1142.77	126.974	14.46	<0.000
X_1_ (Temp.)	1	35.02	35.020	3.99	0.054
X_2_ (Time)	1	213.08	213.083	24.27	0.000
X_3_ (Catalyst)	1	512.24	512.236	58.33	0.000
X_1_^2^	1	1.93	1.931	0.22	0.642
X_2_^2^	1	126.36	126.362	14.39	0.001
X_3_^2^	1	68.81	68.807	7.84	0.008
X_1_X_2_	1	43.85	43.851	4.99	0.032
X_1_X_3_	1	27.33	27.332	3.11	0.086
X_2_X_3_	1	129.34	129.343	14.76	0.000
Residual	35	307.35	8.781		
Lack of fit	3	41.07	13.691	1.65	0.198
Pure error	32	266.27	8.321		
Total	44	1450.11			

**Table 4:** Proximate composition of *Gracilaria verrucosa* and *Gelidium amansii*.

Seaweed	Galactan (%)	AHG (%)	Glucan (%)	Crude protein (%)	Moisture (%)	Ash (%)	Others (%)
*G. verrucosa*	38.53	9.29	9.76	14.63	5.27	4.94	17.58
*G. amansii*	27.25	15.38	19.46	15.36	5.21	4.17	13.17

**Table 5:** Summary of reported LA production from various substrates.

Type	Substrate	Catalyst	Temp. (°C)	Time (min)	LA titer (g/L)	LA yield (wt%)	Reference
Carbohydrate	Glucosamine	5% Amberlyst 15	180	60	18.5^1^	36.9^2^	[27]
		15 mol% ZrOCl_2_	200	20	6.9^1^	13.8^2^	[28]
		0.3 M H_3_NSO_3_	200	15	22.8	18.2^2^	[29]
	Chitosan	0.2 M CH_3_SO_3_H	200	30	5.6^1^	28.2^2^	[30]
	Galactose	0.4 M CH_3_SO_3_H	188	20	15.3	25.5^2^	[31]
	Agarose	1% H_2_SO_4_	180	10	-	45.8^2^	[17]
	Agar	4.5% H_2_SO_4_	180	59	3.5	34.8^3^	In this study
Algae biomass	*Eucheuma spinosum*	0.6 M FeCl_3_	200	30	4.3	11.6^2^	[21]
	*Gracilaria verrucosa*	0.5 M CH_3_SO_3_H	180	20	22.0^1^	36.9^3^	[19]
		6.25% Purolite CT269DR	200	90	22.6	30.3^3^	[20]
		8% H_2_SO_4_	172.5	67.4	3.1	52.9^3^	In this study
					49.8	34.6^3^	
	*Gelidium amansii*	Soaking: 8.97% H_2_SO_4_ Hydrolysis: 3% H_2_SO_4_ (dilution)	Soaking: 76.19 Hydrolysis: 180	Soaking: 49.52 (h) Hydrolysis: 48.22	-	37.6^2^	[18]
		8% H_2_SO_4_	172.5	67.4	3.0	48.4^3^	In this study
					48.6	31.3^3^	
	*Scenedesmus obliquus*	1.875 M CH_3_SO_3_H	180	40	16.75^1^	40.7^3^	[23]
		0.85 M HCl	180	10	6.7^1^	45.6^3^	[32]
		0.8 M H_2_SO_4_	180	40	9.5^1^	39.9^3^	[33]
	Chlorella vulgaris	0.95 M HCl	170	30	5.8^1^	39.3^2^	[34]
		0.5 M CH_3_SO_3_H	195	35	5.8^1^	39.2^2^	[35]
Lignocellulosic biomass	Corncob	1 M H_2_SO_4_	180	30	17.2	19.9^3^	[24]
	Poplar wood	0.95 mol/L *p*-TsOH	162	64	18.4	29.6^2^	[25]

^1^LA titers were back-calculated from the reported data in the corresponding reference.

^2^LA yields were calculated on a weight basis (w/w%) using the LA and carbohydrate amounts derived from the reported information in the corresponding reference.

^3^LA yields were calculated directly on a weight basis (w/w%) using the LA and carbohydrate amounts reported in the corresponding reference.
