# Supplementary material for: High-Yield and High-Titer Production of Levulinic Acid from Red Macroalgae via Sulfuric Acid-Catalyzed Hydrolysis: Optimization by Response Surface Methodology and Application to Actual Biomass
Source: J Microbiol Biotechnol. 2026 Sep 18;36:e2606033. doi: 10.4014/jmb.2606.06033 (PMC13630533; doi:10.4014/jmb.2606.06033)
Supplement: Supplementary file 1 [file jmb-36-e2606033-supple.pdf]

J. Microbiol. Biotechnol. 2026. 36: e2606033

https://doi.org/10.4014/jmb.2606.06033

Supplementary Table and Figure

High-yield and high-titer production of levulinic acid from red macroalgae via sulfuric acid-catalyzed hydrolysis: Optimization by response surface methodology and validation with actual biomass

Table S1. Regression Equation in Uncoded Units for Y_{LA}

$Y_{LA}=-227+2.05T+1.015t+12.04C-0.00417T^2-0.001407t^2-0.2035C^2-0.00390Tt-0.0431TC-0.01914tC$

T, *t*, and *C* represent the uncoded variables for reaction temperature (°C), reaction time (min), and H_2SO_4 cocentration (wt%), respectively.

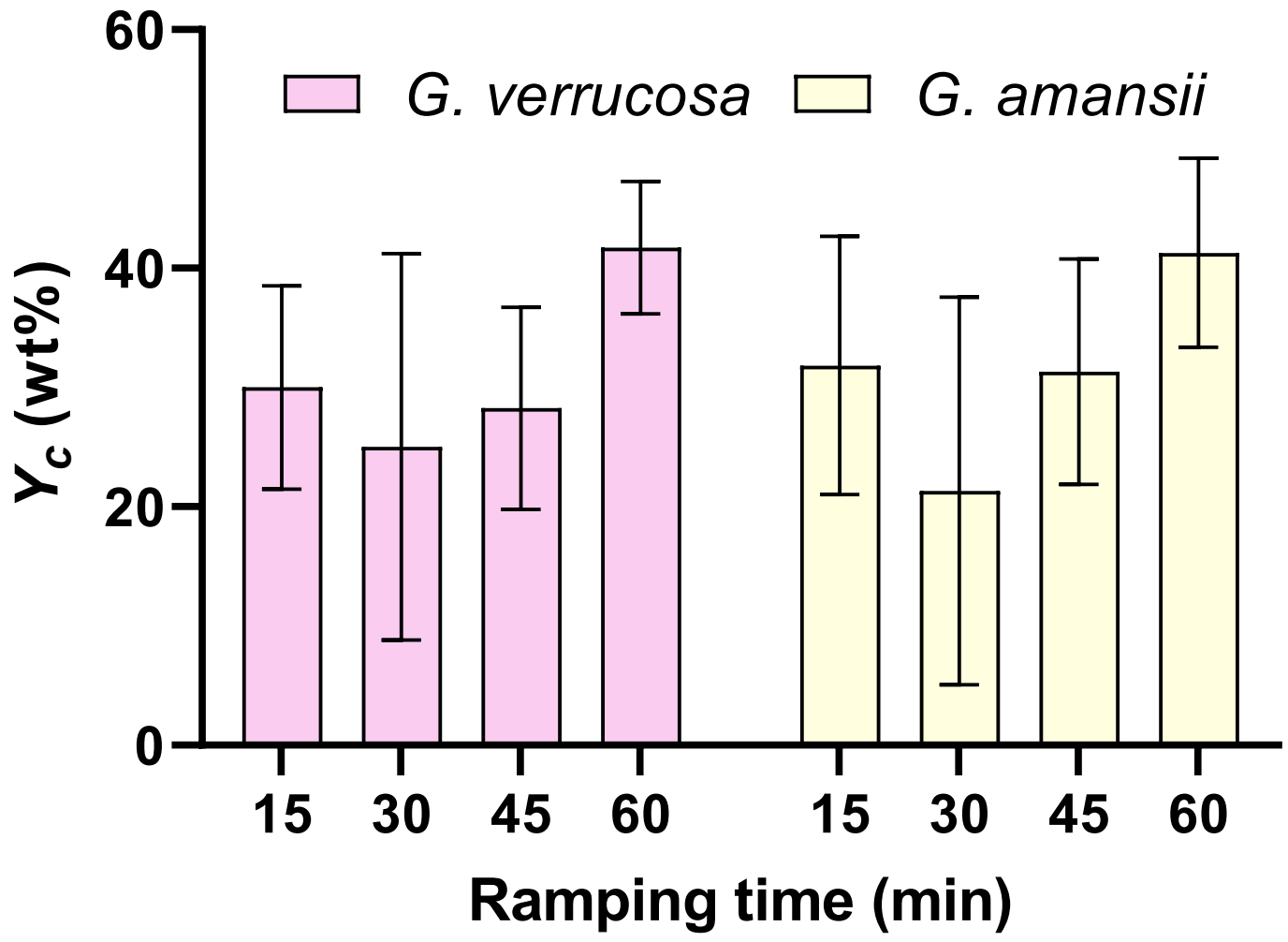

Fig. S1. Effect of different ramping times on LA production from *Gracilaria verrucosa* and *Gelidium amansii*. Reactions were conducted at the optimized conditions (172.5 °C, 67.4 min, 8% H_2SO_4) with a 5% (w/v) substrate concentration.
